# Targeting Progranulin as an Immuno-Neurology Therapeutic Approach

**DOI:** 10.3390/ijms242115946

**Published:** 2023-11-03

**Authors:** Maria A. Boylan, Andrew Pincetic, Gary Romano, Nadine Tatton, Sara Kenkare-Mitra, Arnon Rosenthal

**Affiliations:** Alector, Inc., 131 Oyster Point Blvd, Suite 600, South San Francisco, CA 94080, USA

**Keywords:** PGRN, frontotemporal dementia, immuno-neurology, neuroinflammation, neuroimmunology

## Abstract

Immuno-neurology is an emerging therapeutic strategy for dementia and neurodegeneration designed to address immune surveillance failure in the brain. Microglia, as central nervous system (CNS)-resident myeloid cells, routinely perform surveillance of the brain and support neuronal function. Loss-of-function (LOF) mutations causing decreased levels of progranulin (PGRN), an immune regulatory protein, lead to dysfunctional microglia and are associated with multiple neurodegenerative diseases, including frontotemporal dementia caused by the progranulin gene (*GRN*) mutation (FTD-*GRN*), Alzheimer’s disease (AD), Parkinson’s disease (PD), limbic-predominant age-related transactivation response deoxyribonucleic acid binding protein 43 (TDP-43) encephalopathy (LATE), and amyotrophic lateral sclerosis (ALS). Immuno-neurology targets immune checkpoint-like proteins, offering the potential to convert aging and dysfunctional microglia into disease-fighting cells that counteract multiple disease pathologies, clear misfolded proteins and debris, promote myelin and synapse repair, optimize neuronal function, support astrocytes and oligodendrocytes, and maintain brain vasculature. Several clinical trials are underway to elevate PGRN levels as one strategy to modulate the function of microglia and counteract neurodegenerative changes associated with various disease states. If successful, these and other immuno-neurology drugs have the potential to revolutionize the treatment of neurodegenerative disorders by harnessing the brain’s immune system and shifting it from an inflammatory/pathological state to an enhanced physiological/homeostatic state.

## 1. Introduction

Neurodegenerative disorders are typified by misfolded proteins that accumulate in the diseased brain. For example, Alzheimer’s disease (AD) is defined by the accumulation of extracellular amyloid-β (Aβ) peptides and hyperphosphorylated tau protein that form neurofibrillary tangles (NFTs) inside neurons [1,2], Parkinson’s disease (PD) is characterized by the presence of α-synuclein fibrils that form Lewy bodies [3], and Huntington’s disease (as well as eight additional polyglutamine disorders) is associated with aggregation of the Huntingtin protein due to expansion of a polyglutamine tract within its N-terminal region [4]. Approximately 97 percent of all amyotrophic lateral sclerosis (ALS) cases, as well as ~50 percent of frontotemporal dementia (FTD) cases, are invariably associated with cytoplasmic aggregation of hyperphosphorylated transactivation response deoxyribonucleic acid binding protein 43 (TDP-43) [5,6,7], while the remaining ~3 percent of ALS cases are typified by misfolded superoxide dismutase 1 (SOD1) proteins, and the remaining FTD cases are linked to misfolded and hyperphosphorylated tau inclusions or fused in sarcoma (FUS) inclusions [8,9,10].

Given the strong genetic and anatomical association between misfolded proteins and neurodegeneration, the dominant drug development approaches over the last two decades have focused on the prevention, reversal, or removal of misfolded proteins from the human brain. Thus, multiple drugs that target Aβ [11,12,13], tau [14], α-synuclein [15], Huntingtin [16], *C9orf72* [17,18], SOD1, [19], and TDP-43 [20] are in clinical trials or under development. The relatively recent success of targeting misfolded proteins has led to accelerated approvals, based on data including predictive surrogate biomarkers, of the anti-Aβ monoclonal antibody aducanumab (ADUHELM^®^) [13] for AD by the Food and Drug Administration (FDA) but not by the European Medicines Agency (EMA), and lecanemab (LEQEMBI^®^) [11] for AD as well as an anti-SOD1 drug, tofersen (QALSODY^TM^), for SOD1-ALS [19]. Lecanemab received traditional FDA approval based on clinical efficacy in patients with early AD [11].

An alternative therapeutic strategy to treat degenerative brain disorders targets microglia, which function as brain-specific innate immune cells [21,22,23] to counteract multiple disease pathologies. This strategy, which the authors designate as immuno-neurology, is conceptionally akin to immuno-oncology. There is now an understanding that cancer is a failure of immune surveillance and that instead of targeting cancer cells directly with radiation, chemotherapy, or toxin-conjugated antibodies, one can stimulate and harness the immune system to eradicate tumors. Similarly, the guiding premise of immuno-neurology argues that neurodegeneration is the result of neuroimmune surveillance failure. Physiologically active microglia constantly surveil for and remove pathogens [24], cell and protein debris, protein aggregates, dysfunctional nerve cells, and damaged synaptic nerve connections [25]. Other microglial activities include migrating toward and having contact with leaky blood vessels to support the integrity of the blood–brain barrier (BBB) [26] in damaged brain tissue [27], regulating nerve conduction [28], instructing oligodendrocytes to replace damaged myelin [29], and signaling astrocytes to protect and nourish neurons [30].

The capacity of microglia to orchestrate brain homeostasis by adapting to a context-specific phenotype declines with age [31] due to both the natural senescence process [32] as well as common genetic mutations [33,34,35]. Moreover, as microglia age, their ability to sustain the surveillance, prevention, support, and repair tasks that are essential to homeostasis in the central nervous system (CNS) declines [36]. Microglia can develop a damaging proinflammatory response [37], which leads to a failure to clear misfolded proteins [37] and instead results in injury of surrounding neurons [38] and misleading instructions provided to astrocytes [39] and oligodendrocytes [40,41]. Further, with aging, the microglial response to challenge is often larger and more prolonged [42]. As a result, in certain circumstances, microglia may fail to prevent and may even contribute to the development of neurodegenerative disorders.

**Figure 1:**
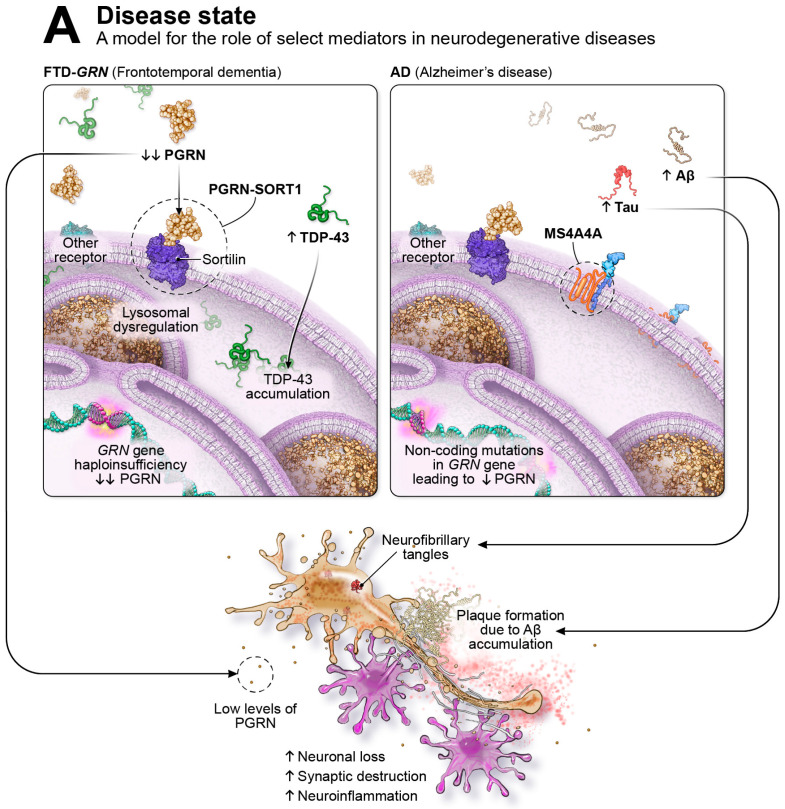

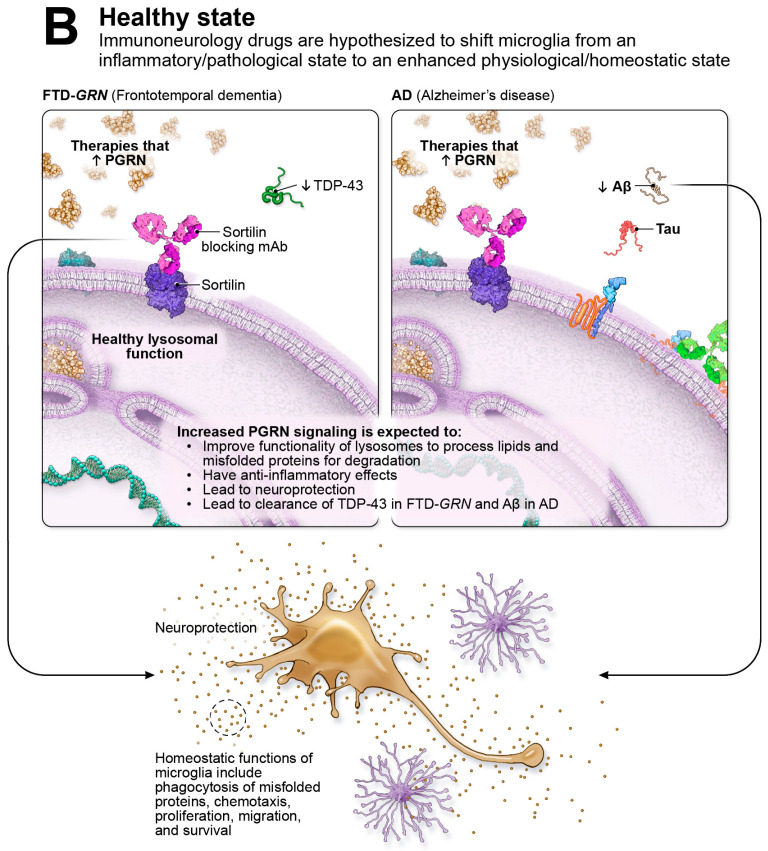
Immuno-neurology Therapeutic Approach. Shown in Figure 1A on the left, in FTD-*GRN*, genetic mutations in the *GRN* gene cause haploinsufficiency and result in substantial PGRN reductions (indicated by the down arrows) leading to various pathological processes, including extra-nuclear TDP-43 accumulation (designated by the up arrow), lysosomal dysfunction, hyperactive microglia, neuronal loss, synaptic destruction, and neuroinflammation. On the right side of Figure 1A, non-coding mutations in the *GRN* gene reduce PGRN levels and increase the risk of neurodegenerative diseases such as PD, ALS, LATE, and, illustrated here, AD. AD is defined by the pathological accumulation of misfolded Aβ forming plaques and tau aggregating into intra-cellular neurofibrillary tangles (NFTs); accumulation of these proteins are highlighted by the up arrows. Additional pathological features of AD include lysosomal dysfunction, hyperactive microglia, neuronal loss, synaptic destruction, and neuroinflammation. Highlighted with dashed-line circles are examples of immune checkpoint-like targets in immuno-neurology, including PGRN-SORT1 and MS4A. Immuno-neurology targets encourage healthy homeostatic functions of microglia, including phagocytosis, chemotaxis, proliferation, migration, and enhanced survival. As illustrated in Figure 1B, immune checkpoint-like targets such as PGRN-SORT1 are thought to shift microglia from an inflammatory/pathological state to an enhanced physiological/homeostatic state. In Figure 1B on the left, in FTD-*GRN*, therapies that elevate PGRN are thought to restore healthy lysosomal function, including degradation of misfolded proteins such as TDP-43; to have anti-inflammatory effects; and to be neuroprotective. In AD, shown in Figure 1B on the right, therapies that elevate PGRN are thought to restore healthy lysosomal function, including degradation of misfolded proteins such as Aβ; to have anti-inflammatory effects; and to be neuroprotective.

Progranulin (PGRN) is a secreted lysosomal chaperone and growth factor implicated in several processes necessary for normal function in the immune and central nervous systems [43,44,45]. In the CNS, PGRN acts as an autocrine and paracrine neurotrophic factor promoting neuronal survival, axonal outgrowth, and functional recovery following nerve injury [46,47,48,49,50]. Though PGRN may elicit distinct neuroimmune modulatory and neurotrophic pathways in microglia and neurons, respectively, its regulation of lysosomal homeostasis is shared across cell types. Transcriptionally, PGRN is regulated by the transcription factor EB (TFEB), a master regulator of lysosomal biogenesis, autophagy, and innate immune activation [51,52,53]. PGRN localizes to lysosomal compartments in microglia, neurons, and other cells, indicating that it can be directed towards the lysosome through intracellular trafficking or endocytosis of extracellular protein. Cells deficient in PGRN display abnormal endolysosomal vacuolization, lysosomal membrane damage, and decreased activity of lysosomal enzymes. In frontotemporal dementia caused by progranulin gene (*GRN*) mutation (FTD-*GRN*), deficits in PGRN lead to pathological processes, including TDP-43 accumulation [54], lysosomal dysfunction, complement activation, neuroinflammation, and astrogliosis, as well as accumulation of neuronal debris [44,54,55], as illustrated in Figure 1A. The absence of PGRN drives age-related changes in microglia, shifting the neuroimmune cells from a healthy to a disease-specific state. Alterations in microglial phenotypes lead to an increase in lysosomal dysfunction and neuroinflammation, heightened production of complement proteins, and intensified synaptic pruning [56,57].

The goal of immuno-neurology is to restore microglia to optimal functionality and alter their phenotype to encourage functions that might combat neurodegenerative processes. “Immune checkpoint–like” genes regulate microglial proliferation, migration, survival, energy generation, lysosomal function, phagocytic ability, and chemotaxis [33], and examples of such targets are highlighted in Figure 1A. At least 20 out of the 84 familial mutations that alter the risk of developing AD are found in genes specifically expressed on or that are enriched in microglia [33,34,35]. Microglial regulators appear as genetic risk for other neurodegenerative diseases, including PD [58], multiple sclerosis (MS) [59], adult-onset leukoencephalopathy with axonal spheroids and pigmented glia (ALSP) [60], and FTD [61], suggesting that failure of neuroimmune surveillance may be attributed to multiple neurodegenerative disorders [33,34,35,61,62,63,64]. The genetic risk genes that regulate microglial functionality provide us with potential molecular levers to manipulate the neuroimmune capacity of microglia in disease settings to create a new category of drugs that can be deployed as monotherapy or in combination with other therapies. Potential therapies designed to elevate PGRN levels in FTD-*GRN* are currently under clinical investigation. Latozinemab (AL001) is a monoclonal antibody that blocks lysosomal degradation of PGRN and chronically elevates PGRN levels by two- to three-fold. Latozinemab is currently being investigated in a pivotal phase III clinical trial (NCT04374136). Other approaches presently being studied in clinical trials include DNL593, a PGRN protein replacement approach that utilizes protein transport vehicle technology to deliver PGRN to the CNS (NCT05262023), and gene therapy drugs, PR006, PBFT02, and AVB-101, designed to deliver a functional copy of the *GRN* gene to the brain (NCT04408625; NCT04747431; NCT06064890). The following review examines the role of microglia in both homeostasis and neuroimmune system failure in neurodegeneration, discusses the biology of PGRN as an immuno-neurology drug target and its role in human disease, and summarizes current clinical stage interventions for FTD-*GRN* in the PGRN space.

## 2. Neuroimmunology in Dementia and Neurodegeneration

### 2.1. Microglial Role in Brain Function

Microglia are resident immune cells within the CNS with a unique ontological origin that differs from peripheral immune cells [65]. Although early investigations into microglial function focused primarily on pathological contexts, it has become exceedingly clear that microglia are essential in all physiological conditions. Indeed, microglia are responsible for maintaining brain homeostasis, neuronal networks, and synaptic plasticity, all of which are key contributors to brain health [66].

In a homeostatic state, microglia exhibit ramified morphology with contact with neurons, astrocytes, and blood vessels, which enables constant surveillance of their environment, including the functional state of synapses [25]. Conversely, in the context of pathology, survival is optimized as microglia shift to an ameboid shape and become highly phagocytic, as well as directionally chemotactic [67]. Microglia form physical barriers to limit damage and engulf pathogens and debris in pathological circumstances [67]. Further, under conditions such as injury, disease, or illness, microglia serve as the primary source of proinflammatory cytokines to mediate the neuroimmune environment, engaging in phagocytosis of misfolded proteins, tissue repair, and recruitment of peripheral immune cells [25].

### 2.2. Alterations in Microglia Function in the Context of Neurodegeneration

Alterations in microglia functionality are implicated in brain aging and neurodegeneration [31,68,69,70]. Accumulation of Aβ and neuronal debris leads to proinflammatory signaling by neurons and astrocytes [71] that shifts microglia from a surveillance state to a phagocytic state [36]. A breakdown in homeostatic neuroimmune interactions occurs with neurodegenerative diseases, although the exact mechanisms are not yet fully elucidated [36]. Within the context of FTD, the *Grn*^−/−^ mouse model, which is devoid of PGRN, shows microglial dysfunction, including impaired phagocytosis and excessive synaptic pruning [56,72]. In FTD patients, microglial activation can be assessed with imaging biomarkers utilizing the ^11^C-PK11195 positron emission tomography (PET) ligand [73], where increased frontotemporal microglial activation has been described in sporadic and genetic forms of FTD [74,75,76]. A recent study suggests that microglial activation precedes cognitive decline and that higher levels of microglial activation are associated with faster longitudinal cognitive decline beyond brain atrophy, which seems to constitute an independent effect [73]. Studies on postmortem human samples report regional microglial activation that differentiates FTD from AD and from controls [77,78,79].

PET measures of microglial activation (^11^C-PK11195) in patients with mild cognitive impairment (MCI) and AD implicate higher, likely aberrant, microglial activation at baseline as a predictor of subsequent longitudinal cognitive decline [76]. Although microglial dynamics in AD remains an area of active investigation, early in the disease process, microglial-mediated degradation and removal of Aβ and tau can be neuroprotective [36,80]. Eventually, excessive proinflammatory cytokines prevent microglia from continued clearance of misfolded and aggregated proteins by altering their phagocytic ability [81], which contributes to their phenotypic shift from neuroprotective to proinflammatory activities [69,70]. This neuroinflammatory shift is evidenced by an increase in the size and number of Aβ plaques as well as hyperphosphorylation and spread of tau pathology later in the disease [82,83,84].

In AD, neuroinflammation contributes to the disease pathophysiology, with alterations in the balance between proinflammatory and anti-inflammatory cytokines [85]. Dysfunction of the endosomal–lysosomal system is one of the key microglial pathologies that emerge in aging and neurodegeneration. This system is essential for the degradation of misfolded proteins, such as Aβ, and as the disease progresses, the endosomal–lysosomal system becomes overwhelmed with internalized misfolded proteins to the point that it can no longer degrade, ultimately leading to a stress response and inability to clear misfolded proteins [86]. Such pathological processes observed in AD are highlighted in Figure 1A on the right side. Abnormalities in endosomal and lysosomal function play key roles in neurodegenerative processes [87,88] and occur early on in AD [89]. PGRN is a critical lysosomal chaperone that is required for lysosomal function and for the ability of microglia to counteract misfolded proteins [90].

Evidence from genome-wide association and other genomic studies consistently identifies genes linked to microglial function, including phagocytosis, as risk factors for AD and related dementias [33,34,35,64,91,92,93]. For example, a risk gene implicated in AD is found within membrane-spanning 4-domains subfamily A (*MS4A*) [64,94], and genes within this cluster are highly expressed on microglia [95]. Haplotypes of the *MS4A* gene that confer lower risk for AD are associated with greater soluble triggering receptor expressed on myeloid cells 2 (sTREM2) in cerebrospinal fluid (CSF) [96] In untreated AD patients, greater sTREM2 has been associated with preservation of cognition and less clinical progression over time [97].

### 2.3. PGRN—A Key Neurotrophic Factor and Regulator in CNS Homeostasis and Immunity

Human PGRN, encoded by *GRN*, is primarily expressed in neurons [98] and microglia [56,99,100,101] in the CNS and is a key regulator of lysosomal function, microglial homeostasis, and anti-inflammatory activity [44,102]. The *GRN* gene is located on chromosome 17q21 and comprises 12 protein-coding exons [103,104]. Upon transcription, 2 adjacent exons spliced together form a granulin domain, which is a conserved motif defined by a characteristic pattern of 12 cysteine residues [105]. The full-length PGRN protein consists of 7.5 tandem repeats of granulin domains separated by short linker sequences, with each domain forming 4 β hairpin structures “stapled” together by 6 parallel disulfide bonds [106]. In some circumstances, the linker sequences are proteolytically cleaved by proteases to produce individual peptides designated granulins A through G.

In the context of immune regulation, PGRN expression is significantly upregulated under various inflammatory conditions, such as tissue injury or autoimmunity, and has been shown to play a protective role in these settings [107]. As a full-length protein, several studies demonstrate that PGRN modulates the effector functions of multiple immune cells. For example, PGRN was shown to suppress the production of cytokines/chemokines and reactive oxygen species (ROS) from activated macrophages and neutrophils. PGRN also promotes the differentiation of regulatory T cells (Tregs) and helps maintain their immunosuppressive properties [108]. The pleiotropic function of PGRN is, in part, attributed to its interactions with multiple receptors and/or binding partners that dictate biological activity. The anti-inflammatory function of PGRN is potentially mediated through its interaction with tumor necrosis factor (TNF) receptors 1 (TNFR1) and 2 (TNFR2) [109]. PGRN inhibits TNF-α induced inflammatory signaling pathways through receptor blockade of TNFR1. Alternatively, PGRN may also trigger a protective, immunosuppressive signaling cascade through its high-affinity interaction with TNFR2 [109,110,111]. More recently, PGRN has been proposed to directly inhibit type IIA secreted phospholipase A2 (sPLA2-IIA), a secreted phospholipase involved in innate immune responses through the generation of lipid mediators of inflammation [112]. Additionally, factors that promote or prevent proteolytic cleavage of PGRN determine the resolution of an inflammatory response. Antibody-mediated inflammatory reactions are compromised in mice deficient in proteinase-3 and neutrophil elastase due to excessive accumulation of PGRN in afflicted tissues [113]. Conversely, mice deficient in secretory leukocyte peptidase inhibitor (SLPI), a soluble factor that shields PGRN from elastase-mediated degradation, present with an impaired wound-healing response due to heightened leukocyte infiltration and inflammation [114]. Whether the proinflammatory response following PGRN cleavage occurs because of the elimination of anti-inflammatory properties of full-length PGRN or due to the activation of inflammatory pathways through granulin peptides remains to be fully elucidated.

Knockdown of PGRN reduces primary neuron survival and neurite outgrowth, while the addition of recombinant PGRN rescues neurons from cell death induced by nerve growth factor withdrawal [115]. Exogenous PGRN protects cortical and motor neurons from toxin- or ischemic-mediated cell death by activating the extracellular-signal-regulated kinase/90 kilodalton ribosomal s6 kinase (ERK/p90RSK) and phosphoinositide 3-kinase/protein kinase B (PI3K/Akt) pathways [116,117]. The identity of the receptor(s) transducing pro-growth or pro-survival signals of PGRN on neurons remains elusive. Sortilin, a member of the vacuolar protein sorting 10 protein (Vps10p) receptor family, is the first receptor identified to bind PGRN with high affinity and mediate endocytic clearance of extracellular PGRN [118]. However, the neurotrophic functions of PGRN occur independently of sortilin, as genetic ablation of this receptor fails to negate PGRN-dependent neuronal survival. PGRN may also affect neuronal health through non-cell autonomous pathways emanating from glial cells. In co-culture systems with induced pluripotent stem cells (iPSC), *GRN*^−/−^ iPSC microglia (iMG) and/or astrocytes enhanced neuron cell death and recapitulated biochemical features of TDP-43 pathology, which could be partially rescued with the addition of recombinant PGRN [119]. Similar observations have been reported in co-culture systems using iMG harboring deleterious mutations in TDP-43 and *C9orf72*, marking glial activation as preceding neuronal loss. Since hyperactivation of glial cells is a characteristic feature of neuroinflammation in several neurodegenerative diseases, the immune-modulatory properties of PGRN may restore glial functionality to maintain and promote brain health.

The mechanism by which PGRN regulates lysosomal function in cells is complex and dynamic. For example, PGRN binds and facilitates lysosomal trafficking of β-glucocerebrosidase (GCase), a lysosomal lipase linked to Gaucher disease and PD [120,121,122,123]. PGRN also interacts with prosaposin (PSAP), another lysosomal regulatory protein involved in glycosphingolipid metabolism [118,124,125]. PGRN/PSAP heterodimers form in the extracellular space where either protein can coordinate endocytic uptake of the protein complex into cells for lysosomal targeting [124]. In the lysosome, PSAP is then cleaved into peptide activators of various lipases, including GCase. In addition to regulation of lipid catabolism, PGRN also regulates lysosomal proteolytic activity linking PGRN deficiency with the accumulation of misfolded proteins. The activity of cathepsin D (CTSD), for example, is decreased in brain lysates obtained from *Grn*^−/−^ mice. However, the addition of recombinant PGRN in lysate fractions effectively restores CTSD activity [46,126,127]. The neuroprotective functions of PGRN in promoting microglial and lysosomal homeostasis highlight the therapeutic potential of exploiting this pathway in diseases with reduced expression or loss of function (LOF) mutations in PGRN.

### 2.4. PGRN’s Role in Human Disease

Microglia that are deficient in PGRN are hyperactive, destroy synaptic connections and nerve endings, secrete toxic mediators [53,56], and induce the aggregation of cytoplasmic TDP-43 in adjacent neurons [57]. In humans, homozygous *GRN* gene mutations invariably lead to neuronal ceroid lipofuscinosis (NCL) with an age of onset <25 years with 100 percent penetrance [128]. NCL is a lysosomal storage disorder that presents in early adulthood with visual impairment, seizures, dementia, and premature death [129]. Over 100 familial heterozygous LOF mutations in the *GRN* gene have been identified that result in 50 percent or less of the normal levels of PGRN and cause FTD-*GRN* before age 70, with penetrance of over 90 percent [130]. LOF mutations in the *GRN* gene cause haploinsufficiency with subsequent frontotemporal lobar degeneration (FTLD) and TDP-43 accumulation. Pathological features of NCL have been recognized in FTD-*GRN*, including lipofuscinosis and indications of intracellular NCL-like storage material [131].

FTD is comprised of a clinically and pathologically heterogeneous spectrum of neurodegenerative disorders, including the behavioral variant (bvFTD) and the language variants, otherwise known as primary progressive aphasia (PPA), that can manifest as either nonfluent/agrammatic variant (nfvPPA) or the semantic variant of PPA (svPPA); a third variant of PPA, the logopenic variant, is typically associated with AD [132,133]. Brain atrophy in FTD is characteristically observed in the frontal and temporal lobes; may be symmetric or asymmetric; and may vary according to the genetic mutation, clinical phenotype [132,133], and regional progression of atrophy [134].

Non-coding mutations in the *GRN* gene that reduce the levels of PGRN by ~10 to 20 percent are associated with an increased risk for AD [64,135,136], PD [137], ALS [138], and limbic-predominant age-related TDP-43 encephalopathy (LATE) [139]. Levels of *GRN* expression were found to be lower in parietal regions of AD patients with the rs5848 *T* allele [136], and in a sample of controls, AD, FTD, and other dementias, homozygous carriers of the rs5848 *T* allele had the lowest levels of serum PGRN [140]. Moreover, missense mutations in *GRN* that lead to interference with PGRN folding and, ultimately, early degradation of the PGRN protein have been identified in individuals with AD [135]. Conversely, overexpression of PGRN is protective in rodent models of FTD [46,141,142], AD [72], PD [143], ALS [46,144], lysosomal storage diseases [145], and arthritis [146]. Taken together, even slight reductions in the level of PGRN protein are associated with increased risk for neurodegenerative processes, providing additional foundation for PGRN as an immuno-neurology drug target.

### 2.5. PGRN Modulation in Animal Models of Neurodegeneration

Initial studies of PGRN deficiency conducted on mice frequently used models that included heterozygous LOF *Grn*^+/−^ and homozygous LOF *Grn*^−/−^. Although *Grn*^+/−^ mouse models do not recapitulate the neuropathology observed in humans with FTD-*GRN*, there is evidence for the social behavioral phenotypes [147]. As a result, researchers often utilize the *Grn*^−/−^ model, with the caveat that humans with FTD-*GRN* do not have a complete loss of the PGRN protein. PGRN-homozygous LOF mice (*Grn*^−/−^) produce less anti-inflammatory but more proinflammatory cytokines than wild-type mice; further, when challenged with infection, PGRN-deficient mice exhibit greater neuroinflammation. Higher activation of microglia and astrocytes, in addition to TDP-43 accumulation, were observed in PGRN-deficient mice with increased age [148]. In the same mouse model, excessively activated microglia accumulated around the site of the injury [53]. In *Grn*^−/−^ mice, PGRN deficiency leads to disrupted lysosomal homeostasis observed first in microglia, followed by other cell types such as neurons and astrocytes [149].

Hyperphosphorylated extranuclear inclusions of the TDP-43 protein have been implicated in FTD and ALS [7,150], AD [151,152], and LATE [153]. Loss of nuclear TDP-43 may lead to dysfunction in RNA splicing, leaving neurons, and in particular their axons, susceptible [154]. Autophagy, the process of breaking down or destroying substances such as proteins, is impaired in neurons deficient in PGRN, ultimately leaving these neurons susceptible to the accumulation of pathological TDP-43 compared to *Grn*^+/+^ neurons [155]. For a high-level overview of the effect of PGRN knockout on neurons, microglia, and astrocytes in FTD-*GRN*, as well as animal and in vitro models of FTD-*GRN*, see Table 1.

In contrast, models of PGRN overexpression seem to be protective. In zebrafish and mouse models, respectively, overexpression safeguards against pathological TDP-43–induced axonopathy [144] and against TDP-43–induced neurodegeneration [46]. Further, fewer activated microglia accumulate in response to injury in a mouse model with PGRN overexpression [156]. Adeno-associated viral vector (AAV)-*GRN* therapy has been studied in *Grn*^−/−^ mice and is associated with improvements in neuronal and microglial PGRN deficiencies [141,157], including microgliosis [158]. Further, systemic delivery of PGRN fused to a TfR-targeting moiety increases CNS levels of PGRN and reduces microglial inflammation and lysosomal dysfunction in *Grn*^−/−^ mice [159]. In addition, *GRN*^−/−^ mature brain organoids developed TDP-43 pathology that was partially rescued with PGRN treatment [119]. Table 1 details the effect of overexpression of PGRN on neurons, microglia, and astrocytes in animal and in vitro models of FTD-*GRN*.

Animal models of amyloidosis and tau allow for investigations into the potential contributions of PGRN to AD pathology and suggest that decreased levels of PGRN play a role in Aβ aggregation, increased tau phosphorylation, and complement activation [72,160,161]. Further, the administration of PGRN enhances microglial and lysosomal clearance of Aβ, leading to fewer Aβ plaques; protects from neuronal loss; and reduces astrogliosis and microgliosis [162,163]. 5xFAD mice, which display high plaque load and neurodegeneration, showed evidence of reduced neuronal loss compared to controls, as well as diminished plaque load, when PGRN was overexpressed using lentiviral vectors, lending additional support for a protective role of PGRN [72]. Lysosomal dysfunction in AD contributes to the accumulation or lack of clearance of Aβ and tau [164,165,166]; TDP-43 has also been found to accumulate in the context of AD [151,152]. However, the mechanisms by which neuronal and microglial PGRN directly contribute to AD pathology continue to be elucidated [167]; Table 1 provides an overview of the current state of the literature examining the effects of PGRN knockout and PGRN overexpression on neurons, microglia, and astrocytes in AD animal models. Dysfunctional lysosomal activity may be a possible common mechanism for degenerative brain disorders, and therapies that enhance or repair lysosomal function in the aged brain, such as PGRN, may have broad therapeutic utility.

**Table 1:** Effect of modulation of PGRN in neurons, microglia, and astrocytes.

	PGRN Insufficiency in FTD-*GRN*	PGRN Knockout in FTD-*GRN* Animal Models/ In Vitro	Administration of PGRN in FTD-*GRN* Animal Models/ In Vitro	PGRN Knockout in Animal Models of AD/In Vitro	Administration of PGRN in AD Animal Models/In Vitro
Neurons	Neuronal loss; downregulation of synaptic genes; overlap with TDP-43 target RNAs [168]; cytoplasmic TDP-43 deposition [142,168,169]; tau accumulation [170]	Susceptible to apoptosis/neuronal loss [47,171,172,173,174]; hyperexcitability [175]; reduced dendritic length and reduced spine density [176]; reduction in primary neuron survival and neurite outgrowth [48,115]; lysosomal dysfunction [127,149,155,177,178]; lipofuscinosis [127,172]; results in hyperphosphorylated TDP-43/TDP-43 accumulation [119,127,142,148,155,171,178,179]; disruption of autophagy–lysosomal system [155,171]; CTSD accumulation [142]	Promotes neuronal survival and enhances neurite outgrowth in cultured neurons [50]; rescues neurons from cell death/degeneration [46,115,159,174,179]; protects cortical and motor neurons from toxin- or ischemic-mediated cell death [116]; functions as neurotrophic factor [47,48,144,177,180,181,182]; reverses social dominance deficits and corrects lysosomal dysfunction [157]; rescues TDP-43 LOF [119]; safeguards against pathological TDP-43–induced axonopathy [144]; protects against TDP-43–induced neurodegeneration [46]; reduces TDP-43 phosphorylation [174,179]; accelerates axonal regrowth [156]; stimulates phosphorylation of glycogen synthase kinase-3 beta (GSK-3β) [47]	Increases tau pathology and complement activation [161]	Protective against neuronal loss [72]; reduces synaptic loss [163]
Microglia	Microgliosis [168,169], upregulation of C1q, complement protein; myelin debris accumulation [168]; Myelin loss and white matter pathology [169]	Hyper-inflammatory phenotype [56,148,156,171,172,173,174,178,183]; impairs phagocytosis and motility [72,175]; increases synaptic pruning [56]; lysosomal dysfunction [53,56,149,174,178]; disruption of autophagy–lysosomal system and lipofuscinosis [171]; induces aggregation of TDP-43 in adjacent neurons [57]; increases lysosomal protein and gene expression (LAMP1 and CTSD_mat_) [142]	Reduces microgliosis [141,179]; suppresses CTSD_mat_ [142]; rescued oxidative stress, lysosomal dysfunction, microgliosis, and endomembrane damage [159]; increases number of microglial processes, indicating reduced activation [179]	Increases microgliosis, impairs phagocytosis, and increases plaque load; causes deficits in spatial learning [72]; enhances microglial phagocytosis [161]	Enhances endocytosis of Aβ [72]; reduces microgliosis [72,163]; enhances microglia phagocytosis and co-localization with Aβ [162]
Astrocytes	Disease-specific transcriptional profile; increased synaptic pruning; myelin debris accumulation; disruption of synapse number and morphology [168]; astrogliosis and white matter damage [169]; tau accumulation [170]	Increases astrogliosis [148,171,172,183,184]; disrupts autophagy–lysosomal system [149,171,178]; promotes synaptic degeneration, neuronal stress, and TDP-43 proteinopathy [168]; contributes to BBB disruption [185]	Attenuates pro-inflammatory activation of astrocytes [184]; decreased glial fibrillary acidic protein (GFAP) intensity [179]; number of astrocytes decreased [174]	No studies identified	Reduces astrogliosis [163]

Evidence suggests that neurons and microglia express PGRN, with microglial PGRN depending upon the activation state with higher PGRN levels in response to injury [101,120,186,187]. PGRN expression by astrocytes, on the other hand, is less understood, with some evidence for and against these cells expressing PGRN [101,186]. Table 1 describes the effect of PGRN deficiency on neurons, microglia, and astrocytes during PGRN insufficiency in FTD-*GRN*; the effect of PGRN knockout in FTD-*GRN* animal models or in vitro, when PGRN is administered to animal models of FTD-*GRN* or in vitro; in the case of PGRN reduction in the context of Alzheimer’s Disease, when PGRN is knocked out of AD animal models or in vitro; and finally, when PGRN is administered to AD animal models or in vitro.

### 2.6. Therapeutic Modulation of PGRN

The current clinical-stage therapeutic landscape for FTD-*GRN* encompasses three approaches to elevating PGRN that are being studied in humans thus far: antibody therapy to block sortilin, gene therapy, and PGRN protein-replacement therapy. PGRN-associated therapies, including approaches currently in the preclinical phase, have been reviewed in detail elsewhere [147]. Latozinemab, a human recombinant anti-human sortilin IgG1 monoclonal antibody, is the only therapy currently being studied in a pivotal phase III trial for FTD-*GRN*. Latozinemab blocks the interaction between sortilin and PGRN and decreases the surface expression of sortilin, preventing degradation of PGRN and ultimately elevating extracellular PGRN levels. Importantly, although sortilin is a lysosomal trafficking pathway for PGRN, there are additional independent yet complementary pathways for PGRN to be delivered to the lysosome, namely, PSAP via the cation-independent mannose 6-phosphate receptor (M6PR) and low-density lipoprotein receptor-related protein 1 (LRP1) [124]. PSAP combined with a modified PGRN protein that cannot bind sortilin allows for the delivery of PGRN from the extracellular space to the lysosome [124]. Further, when M6PR or LRP1 functionality is removed, PGRN trafficking is reduced, highlighting the necessary interactions between PSAP, M6PR, and LRP1 in trafficking PGRN to the lysosome. Moreover, ablation of the sortilin receptor in mice does not result in lysosomal dysfunction, microgliosis, or neurodegeneration [118], while M6PR [188], LRP1 [189], and PSAP [190] deficiencies in mice elicit glial and neuronal impairments [118]. Further, in preclinical models, the highest dose of latozinemab at 200 mg/kg did not elicit adverse effects [191]. Taken together, there are alternative pathways for PGRN to traffic to the lysosome beyond sortilin; the interaction between PGRN and PSAP through the M6PR and LRP1 pathways allows for PGRN delivery to the lysosomes of neurons and microglia in support of healthy lysosomal function. Lastly, sortilin is not required for PGRN’s neuroprotective effects [182], lending further support to the proposed mechanism of latozinemab. In a phase I, first-in-human study, a single administration of latozinemab resulted in reduced white blood cell (WBC) sortilin, increased plasma PGRN by three-fold, and increased CSF PGRN by two-fold in healthy volunteers. In at-risk FTD-*GRN* mutation carriers, a single administration of latozinemab restored PGRN to physiological levels [191]. Latozinemab is also being studied in an open-label, phase 2 clinical trial in FTD-*C9orf72*, a genetic mutation that is causal for FTD (INFRONT-2; NCT03987295).

Several other therapies targeting PGRN elevation are also being explored in earlier stages of clinical testing, including phase I/II open-label studies of gene therapy for FTD-*GRN* in the PROCLAIM, upliFT-D, and ASPIRE-FTD clinical trials. In the PROCLAIM study, PR006 is an investigational gene therapy that utilizes adeno-associated viral vector serotype 9 (AAV9) and is designed to deliver a functional copy of the *GRN* gene to the brain (NCT04408625). In the upliFT-D study, PBFT02 is a gene-replacement therapy that employs adeno-associated viral vector serotype 1 (AAV1; NCT04747431); PBFT02 and PR006 are both delivered as a single dose via intra cisterna magna administration. AVB-101, being studied in the ASPIRE-FTD clinical trial, is also an investigational gene-replacement therapy that utilizes AAV9 and intrathalamic delivery of the *GRN* gene (NCT06064890). Gene therapy, although promising due to its single-dose administration, inevitably faces challenges, including the requirement of invasive administration. With regard to dosing, it remains to be seen whether a single dose will lead to a sufficient therapeutic effect, whether the effect will be durable, and even what the appropriate dosage level should be [192]. A further potential difficulty is the adaptive and innate immune systems, which may produce neutralizing antibodies to the viral vector, limiting the therapy to a single administration [145]. This underscores the importance of selecting the appropriate dosage level, which can be partially circumnavigated by giving immunosuppressants, utilizing different vectors for gene delivery, and administering the lowest dose possible [193]. An additional consideration is ensuring adequate viral spread from the injection site; transduction varies according to AAV serotype, with AAV1 and AAV9 associated with neural and glial transduction when administered via intraparenchymal injection [192]. However, CNS cell tropism also varies according to the AAV preparation method, necessitating cell-type-specific promoters. Further, particular AAV serotypes are more likely to undergo axonal transport, which could be essential for the vector to spread beyond the injection site [192]. Extensive details surrounding gene therapies in development for FTD-*GRN* have not been shared beyond the AAV serotype; however, such details, as discussed above, are fundamental to the potential therapeutic effect.

An alternative approach is to deliver the PGRN protein systemically; a phase I/II, placebo-controlled study is evaluating protein transport vehicle technology (DNL593) to allow intravenously administered PGRN protein to move across the BBB and into the CNS (NCT05262023). DNL593 is made up of the PGRN protein fused to an antibody segment that binds to the transferrin receptor, whereby transcytosis facilitates the transfer of DNL593 across the BBB [159]. Regarding protein-replacement therapy, factors worth considering include a short half-life, potential association with autoimmunity [194,195,196,197], the risk of overexpression of PGRN in the periphery and potential off-target effects [198,199,200], BBB permeability, and the possibility that the fusion protein may affect processing of PGRN in the lysosome. Given the systemic route of administration, it is possible that the protein may be metabolized or cleared before it can enter the CNS [201].

Considerations for the class of PGRN elevating drugs include determining long-term safety and the appropriate level of PGRN elevation necessary for clinical, patient, and care partner benefit. CNS and systemic delivery each contend with challenges regarding the dosing schedule, with gene therapies facing invasive procedures while systemically administered agents confront chronic dosing. There is much to be learned from the ongoing clinical programs, including the potential effects of PGRN elevation outside of the CNS.

Although clinical trials investigating PGRN-elevating therapies initially focused on the FTD-*GRN* space, as reviewed in this article, there is promise for PGRN elevation in AD. Indeed, a phase 2 placebo-controlled clinical trial, PROGRESS-AD, plans to investigate a PGRN-elevating monoclonal antibody, AL101 (GSK4527226), in patients with MCI and mild dementia due to AD (NCT06079190).

## 3. Conclusions

Immuno-neurology drugs have the potential to bring about a revolutionary shift in the treatment of degenerative brain disorders, similar to the transformation of cancer treatment by leveraging the immune system to combat tumors. The concept behind immuno-neurology is to harness the power of the immune system to target and address the underlying mechanisms of neurodegeneration. By modulating immune responses and promoting neuroprotective functions, immuno-neurology drugs have the potential to slow disease progression, reduce inflammation, and preserve neuronal health. This emerging field holds great promise for conditions such as AD, PD, and other degenerative brain disorders where immune dysregulation and neuroinflammation play significant roles. As research and clinical trials progress, immuno-neurology drugs could revolutionize the treatment landscape and offer new hope for patients and their families. Further, immuno-neurology is a strong alternative yet potentially complementary approach to current investigative neurodegenerative therapies that primarily focus on removing singular types of misfolded proteins from the CNS. One particularly exciting, genetically validated target in this field is PGRN, a protein that is predominantly expressed in neurons and microglia within the CNS. PGRN plays a crucial role in regulating lysosomal function, microglial homeostasis, and anti-inflammatory responses. Figure 1B explores the potential implications of the immuno-neurology approach to elevating PGRN in the FTD-*GRN* and AD disease states. Clinical trials are currently underway to investigate potential therapies that aim to elevate PGRN levels, offering a potential new avenue for treating neurodegenerative diseases. FTD-*GRN*, a genetically driven form of neurodegeneration, results in a range of pathological processes, such as TDP-43 accumulation, lysosomal dysfunction, neuroinflammation, complement activation, astrogliosis, and the buildup of neuronal debris. The field of immuno-neurology holds significant promise in addressing both genetically validated and sporadic neurodegenerative diseases, as the innate immune system plays a central role in maintaining neuronal health.

## Abbreviations

Abbreviation	Definition
AAV	Adeno-associated viral vector.
AAV1	Adeno-associated viral vector serotype 1.
AAV9	Adeno-associated viral vector serotype 9.
AD	Alzheimer’s disease.
ALS	Amyotrophic lateral sclerosis.
ALSP	Adult-onset leukoencephalopathy with axonal spheroids and pigmented glia.
Aβ	Amyloid-β.
BBB	Blood–brain barrier.
bvFTD	Behavioral variant frontotemporal dementia.
CNS	Central nervous system.
CSF	Cerebrospinal fluid.
CTSD	Cathepsin D.
ERK/p90RSK	Extracellular-signal-regulated kinase/90 kiladalton ribosomal s6 kinase.
FTD	Frontotemporal dementia.
FTD-*GRN*	Frontotemporal dementia caused by progranulin gene mutation.
FTLD	Frontotemporal lobar degeneration.
FUS	Fused in sarcoma.
GCase	β -glucocerebrosidase.
GRN	Progranulin gene.
iMG	iPSC microglia.
iPSC	Induced pluripotent stem cells.
LATE	Limbic-predominant age-related transactivation response deoxyribonucleic acid binding protein 43 encephalopathy.
LOF	Loss of function.
LRP1	Low-density lipoprotein receptor-related protein.
M6PR	Mannose 6-phosphate receptor.
MCI	Mild cognitive impairment.
MS	Multiple sclerosis.
MS4A	Membrane-spanning 4-domains subfamily A.
NCL	Neuronal ceroid lipofuscinosis.
NFT	Neurofibrillary tangles.
nfvPPA	Non-fluent variant primary progressive aphasia.
PD	Parkinson’s disease.
PET	Positron emission tomography.
PGRN	Progranulin.
PI3K/Akt	Phosphoinositide 3-kinase/protein kinase B.
PPA	Primary progressive aphasia.
PSAP	Prosaposin.
ROS	Reactive oxygen species.
SLPI	Secretory leukocyte peptidase inhibitor.
SOD1	Superoxide dismutase type 1.
sPLA2-IIA	Type IIA secreted phospholipase A2.
svPPA	Semantic variant primary progressive aphasia.
TDP-43	Transactivation response deoxyribonucleic acid binding protein 43.
TFEB	Transcription factor EB.
TNF	Tumor necrosis factor.
TNF-α	Tumor necrosis factor alpha.
TNFR1	Tumor necrosis factor receptor 1.
TNFR2	Tumor necrosis factor receptor 2.
Tregs	Regulatory T cells.
sTREM2	Soluble triggering receptor expressed on myeloid cells 2.
TREM2	Triggering receptor expressed on myeloid cells 2.
Vps10p	Vacuolar protein sorting 10 protein.
WBC	White blood cell.
